# Trends in the Proportion of Young Women and Girls Prescribed Spironolactone

**DOI:** 10.1001/jamanetworkopen.2025.0842

**Published:** 2025-03-17

**Authors:** Sarah E. Soppe, Whitney R. Robinson, Mark P. Lachiewicz, Mollie E. Wood

**Affiliations:** 1 Department of Epidemiology, Gillings School of Global Public Health, The University of North Carolina at Chapel Hill; 2 Department of Obstetrics and Gynecology, Duke University School of Medicine, Durham, North Carolina

## Abstract

This cohort study evaluates changes in the proportion of young women and girls prescribed spironolactone from 2000 to 2020.

## Introduction

Spironolactone is approved by the US Food and Drug Administration for cardiovascular disease, with clinical trials primarily evaluating drug safety among older adults with heart failure.^1,2^ However, it is also widely prescribed for androgen-related conditions such as acne in young women and girls, frequently requiring a higher dose than cardiovascular uses (100-200 mg/d vs 25 mg/d).^1^ To address the lack of trial data on higher doses in younger women, some safety issues have been evaluated through observational studies.^3^ However, concerns of vulvar pain from spironolactone-induced androgen deprivation have been raised in a case series,^4^ a potential safety concern not yet evaluated on a population scale. Given its established widespread use for acne,^5^ including among adolescents with increased susceptibility to hormonal impacts, targeted safety studies may be needed. However, the total proportion of younger women and girls using spironolactone across its broad range of indications is currently unknown. To inform the need for additional safety studies in this population, we assessed prescribing trends from 2000 to 2020 stratified by age.

## Methods

This cohort study was approved by the University of North Carolina at Chapel Hill’s institutional review board and did not require patient consent due to use of deidentified insurance claims data. Following Strengthening the Reporting of Observational Studies in Epidemiology (STROBE) reporting guidelines, we constructed a cohort of female patients aged 12 to 40 years in the Merative MarketScan Commercial Claims and Encounters Database. Inclusion criteria included 3 or more months of continuous enrollment with drug coverage and no prior spironolactone prescription fill (eFigure in Supplement 1). New prescriptions were identified with National Drug Codes and defined as prescription fills after 3 months without fills, with adjustment to 6 months in a sensitivity analysis (eTable 1 in Supplement 1). Starting April 2000, age-stratified incidence among all covered lives was calculated for each month and standardized to the region distribution of January 2010, averaging monthly incidences across each year. Indications for use were assessed with *International Statistical Classification of Diseases and Related Health Problems, Ninth *and *Tenth Revision* codes within 30 days before to 3 days after the first prescription fill (eTable 2 in Supplement 1). Analyses were conducted using SAS version 9.4 (SAS Institute) from January to October 2024.

## Results

A total of 38 million met inclusion criteria; of those, 451 234 (1.2%) initiated spironolactone with 51 821 (11.5%) aged 12 to 18 years at initiation (Table). A total of 248 061 (55.5%) were diagnosed with acne, followed by hirsutism (37 496 patients [8.3%]) and polycystic ovary syndrome (36 628 patients [8.1%]); 27 440 (6.1%) had multiple indications. Hypertension and congestive heart failure were rare (17 952 patients [4.0%] and 3679 patients [0.8%], respectively), and 132 634 (29.4%) lacked any of these diagnoses. Requiring 6 months of coverage before prescription fill did not substantially change these distributions. New prescriptions increased from a standardized monthly mean (SD) of 17 (1.2) per 100 000 covered individuals in 2000 to 88 (3.7) per 100 000 in 2020 (Figure). The greatest rise was among those aged 19 to 25 years, though considerable increases were observed for all age groups.

**Table.** Spironolactone Initiator Demographics and Indications for Use Among Young Women and Girls Aged 12 to 40 Years From 2000 to 2020 in the Merative MarketScan Commercial Claims and Encounters Database

Characteristic	No. (%) (N = 451 234)
Age at initiation, y	
12-18	51 821 (11.5)
19-25	131 109 (29.1)
26-32	122 009 (27.0)
33-40	146 295 (32.4)
Region	
North Central	99 226 (22.0)
Northeast	63 123 (14.0)
South	197 920 (43.9)
West	87 287 (19.3)
Unknown	3678 (0.8)
Year of initiation	
2000-2005	24 570 (5.4)
2006-2010	77 764 (17.2)
2011-2015	153 102 (33.9)
2016-2020	195 798 (43.4)
Indication^a^	
Acne	248 061 (55.0)
Hirsutism	37 496 (8.3)
Polycystic ovary syndrome	36 628 (8.1)
Hypertension	17 952 (4.0)
Hidradenitis suppurativa	4490 (1.0)
Congestive heart failure	3679 (0.8)
>1 of These	27 440 (6.1)
None of these	132 634 (29.4)

^a^ Participants were allowed to have more than 1 indication for use if they had multiple *International Statistical Classification of Diseases and Related Health Problems* codes during the assessment window.

**Figure.**
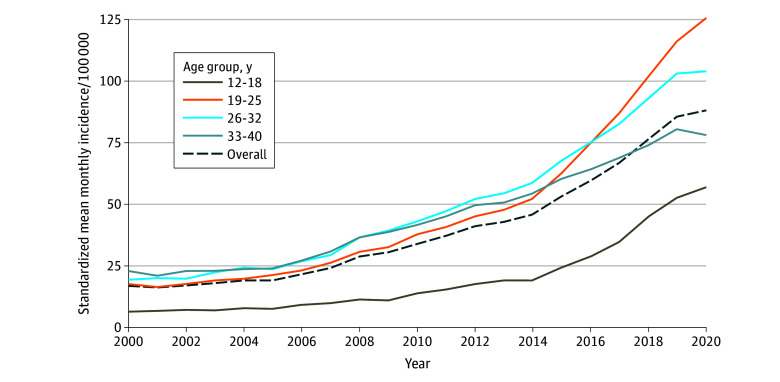
Standardized Mean Monthly Incidence of Spironolactone Stratified by Age at Initiation Estimates are standardized to the region distribution of the study population in January 2010.

## Discussion

We estimate 1.2% of insured young women and girls initiated spironolactone, with this proportion rising considerably in recent years. Consistent with prior studies,^5^ many prescriptions were associated with acne. However, this condition represented only a portion of total use. Most initiators had androgen-related conditions, indications frequently requiring higher doses than those examined in clinical trials for cardiovascular disease.^1,2^ Limitations of this analysis included potential misclassification of indication, as some diagnoses are more likely to be recorded due to better insurance reimbursement. Nonetheless, we expect minimal misclassification across cardiovascular and androgen-related categories. A total of 29.4% lacked a diagnosis for the examined conditions; these patients may have been prescribed spironolactone to manage ascites and edema-related symptoms during cancer treatment,^6^ an indication that could not be incorporated without cancer registry linkage. Despite these limitations, our findings indicate spironolactone among young women and girls is notable and growing, with the great majority having indications requiring higher doses than those examined in trials. Further investment in safety studies may be warranted.

## Notes

Supplement 1. **eFigure.** Exclusion Criteria CONSORT Diagram **eTable 1. **Spironolactone Initiator Demographics and Indications for use in Sensitivity Analysis Requiring 180 Days of Continuous Enrollment Before the Date of First Prescription Fill **eTable 2.*** International Classification of Disease-Clinical Modification, Version 9* (April 1, 2000 – October 31, 2015) and *Version 10* (November 1, 2015- December 31, 2020) Codes, Used to Identify Spironolactone Indications

Supplement 2.
Data Sharing Statement
