# Supplementary material for: Trends in the Proportion of Young Women and Girls Prescribed Spironolactone
Source: JAMA Netw Open. 2025 Mar 17;8(3):e250842. doi: 10.1001/jamanetworkopen.2025.0842 (PMC11915057; doi:10.1001/jamanetworkopen.2025.0842)
Supplement: Supplement 1. — eFigure. Exclusion Criteria CONSORT Diagram eTable 1. Spironolactone Initiator Demographics and Indications for use in Sensitivity Analysis Requiring 180 Days of Continuous Enrollment Before the Date of First Prescription Fill eTable 2. International Classification of Disease-Clinical Modification, Version 9 (April 1, 2000 – October 31, 2015) and Version 10 (November 1, 2015- December 31, 2020) Codes, Used to Identify Spironolactone Indications [file jamanetwopen-e250842-s001.pdf]

Supplemental Online Content

Soppe SE, Robinson WR, Lachiwicz MP, Wood ME. Proportion of young women and girls prescribed spironolactone from 2000 to 2020. *JAMA Netw Open*. 2025;8(3):e250842.
doi:10.1001/jamanetworkopen.2025.0842

eFigure. Exclusion Criteria CONSORT Diagram

eTable 1. Spironolactone Initiator Demographics and Indications for use in Sensitivity Analysis Requiring 180 Days of Continuous Enrollment Before the Date of First Prescription Fill

eTable 2. *International Classification of Disease-Clinical Modification, Version 9* (April 1, 2000 – October 31, 2015) and *Version 10* (November 1, 2015- December 31, 2020) Codes, Used to Identify Spironolactone Indications

This supplemental material has been provided by the authors to give readers additional information about their work.

© 2025 Soppe SE et al. *JAMA Network Open*.

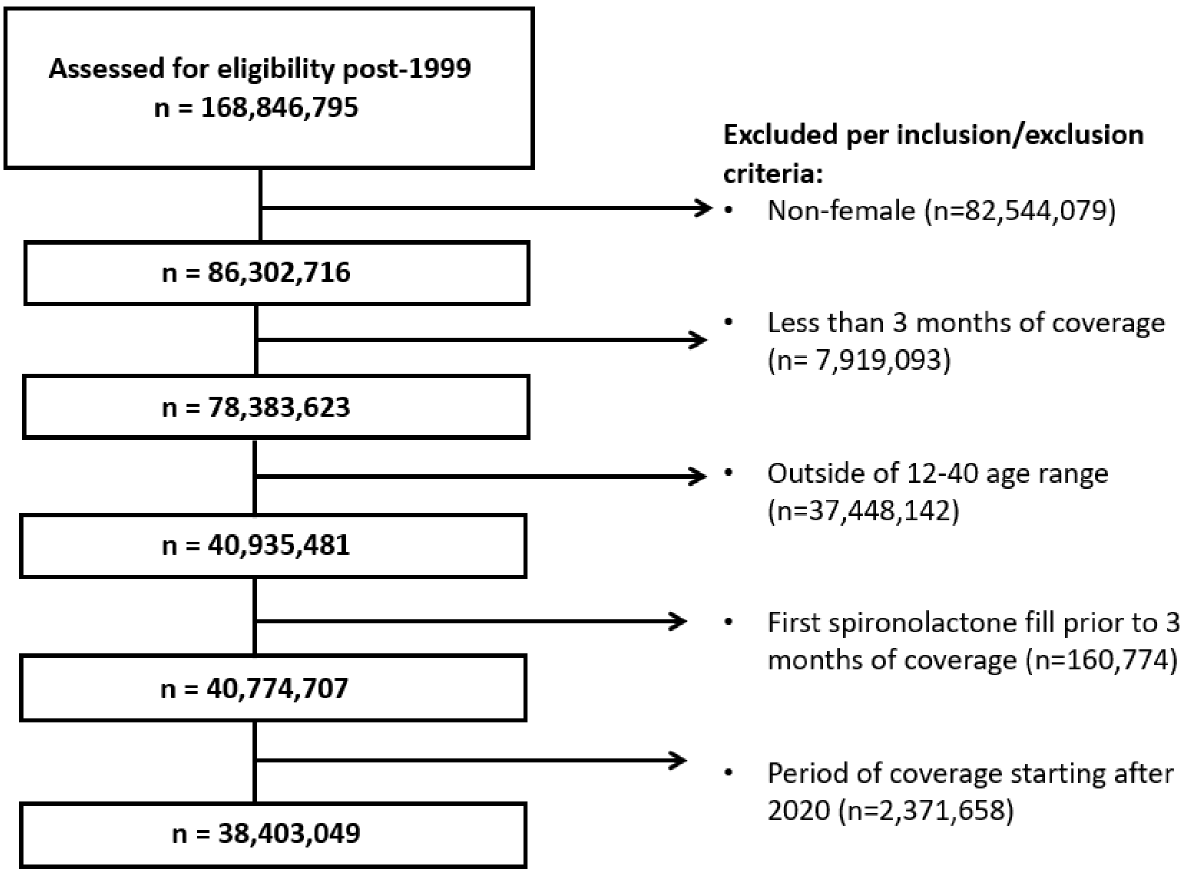

eFigure 1. Exclusion criteria CONSORT diagram.

© 2025 Soppe SE et al. *JAMA Network Open*.

	N (%)
**Total**	385,791 (100.0)
**Age at initiation**	
**12-18**	47,559 (12.3)
**19-25**	113,895 (29.5)
**26-32**	99,239 (25.7)
**33-40**	125,098 (32.4)
**Region**	
**North Central**	85,542 (22.2)
**Northeast**	53,537 (13.9)
**South**	169,289 (43.9)
**West**	74,363 (19.3)
**Unknown**	3,060 (0.8)
**Year of initiation**	
**2000-2005**	19,613 (5.1)
**2006-2010**	65,074 (16.9)
**2011-2015**	131,296 (34.0)
**2016-2020**	169,808 (44.0)
**Indication**[a]	
**Acne**	220,174 (57.1)
**Hirsutism**	32,704 (8.5)
**Polycystic ovarian syndrome**	31,173 (8.1)
**Hypertension**	15,444 (4.0)
**Hidradenitis suppurativa**	4,002 (1.0)
**Congestive heart failure**	3,162 (0.8)
**More than one of these**	23,878 (6.2)
**None**	104,980 (27.2)

eTable 1. Spironolactone initiator demographics and indications for use in sensitivity analysis requiring 180 days of continuous enrollment prior to the date of first prescription fill.

a. Participants were allowed to have more than one indication for use if they had multiple International Classification of Diseases codes during the assessment window.

© 2025 Soppe SE et al. *JAMA Network Open*.

Condition	Diagnostic codes
**Acne**	ICD-9: 706.0, 706.1<br>ICD-10: L70.0, L70.8, L70.9
**Polycystic ovarian syndrome (PCOS)**	ICD-9: 256.4<br>ICD-10: E28.2
**Hirsutism**	ICD-9: 704.00, 704.1<br>ICD-10: L65.9, L68.0
**Hidradenitis suppurativa**	ICD-9: 705.83<br>ICD-10: L73.2
**Hypertension**	ICD-9: 401.xx, 402.xx, 403.xx, 404.xx<br>ICD-10: I10, I11, I12, I13
**Congestive heart failure**	ICD-9: 398.9, 402.01, 404.01, 404.03, 414.8, 425.2, 425.4, 425.5, 425.8, 425.9, 428<br>ICD-10: I09.9, I11.0, I13.0, I13.2, I25.5, I42.0, I42.5-I42.9, I43.xx, I50.x

eTable 2. International Classification of Disease-Clinical Modification codes, version 9 (April 1, 2000 – October 31, 2015) and version 10 (November 1, 2015- December 31, 2020), used to identify spironolactone indications.

© 2025 Soppe SE et al. *JAMA Network Open*.
