# Supplementary material for: Trends in the Proportion of Young Women and Girls Prescribed Spironolactone
Source: JAMA Netw Open. 2025 Mar 17;8(3):e250842. doi: 10.1001/jamanetworkopen.2025.0842 (PMC11915057; doi:10.1001/jamanetworkopen.2025.0842)
Supplement: Supplement 2. — Data Sharing Statement [file jamanetwopen-e250842-s002.pdf]

# Data Sharing Statement

Soppe. Proportion of Young Women and Girls Prescribed Spironolactone From 2000 to 2020. *JAMA Netw Open*. Published March 17, 2025. doi:10.1001/jamanetworkopen.2025.0842

**Data**
**Data available:** No

**Additional Information**
**Explanation for why data not available:** Data contains protected health information.
